# Wearing face masks and possibility for dry eye during the COVID-19 pandemic

**DOI:** 10.1038/s41598-022-07724-0

**Published:** 2022-04-13

**Authors:** Qian Fan, Minhong Liang, Wenjun Kong, Wei Zhang, Hongxia Wang, Jie Chu, Xin Fang, Yi Song, Wenjing Gao, Yan Wang

**Affiliations:** 1 Tianjin Eye Hospital, Tianjin Key Lab of Ophthalmology and Visual Science, Tianjin Eye Institute, Nankai University Affiliated Eye Hospital, Tianjin, China; 2 Nankai Eye Institute, Nankai University, Tianjin, China; 3 Clinical College of Ophthalmology, Tianjin Medical University, Tianjin, 300020 China; 4 Shanghai Hongkou Center for Disease Control and Prevention, Shanghai, China; 5 Beijing You’an Hosptial, Capital Medical University, Beijing, China; 6 Shanghai Guanghua Integrated Traditional Chinese and Western Medicine Hospital, Guanghua Hospital Affiliated to Shanghai University of Traditional Chinese Medicine, Shanghai, China; 7 Department of Ophthalmology, West Hospital of Hetian People’s Hospital, Xinjiang, China; 8 Huainan Experimental Middle School, Anhui, China

**Keywords:** Public health, Epidemiology

## Abstract

This population-based observational, cross-sectional, and descriptive survey was to investigate the relationship of increased face mask usage in the coronavirus disease (COVID-19) era with mask-associated dry eye (MADE). Participants aged 6–79 years old with formal school education were selected. All participants finished the 19-item questionnaire online, distributed through different social media platforms. From 6925 participants who submitted eligible questionnaires, MADE was reported in 547 participants, which included 419 participants who developed new dry eye symptoms after wearing face masks and 128 participants whose pre-existing dry eye symptoms worsened with mask wearing. Longer time of face mask wearing, nonstandard wearing of face masks, reduced outdoor time, decreased daily reading time, shortened visual display terminals time, and dry environment were positively associated with MADE. There were significant associations between perceived MADE and age, female sex, education, use of glasses and contact lenses, and pre-existing dry eye. MADE was more common in adults aged > 20 years than those aged ≤ 20 years or juveniles. MADE incidence increased. Standard wearing of face masks was suggested as a protective factor for MADE. Awareness about the possible risk of MADE should also be created and the clinical dry eye signs should be verified.

Clinical trial registration number: NCT04744805.

## Introduction

The Coronavirus Disease 2019 (COVID-19) has appeared in December 2019 and has been characterized as the first pandemic caused by a coronavirus and is under intense global scrutiny^1^. In order to contain the spread of COVID-19, widespread physical separation measures and movement limitations were implemented by many governments and authorities all over the world^2–4^. COVID-19 outbreak has had a long-term impact on people's lives, including education, business, and the economy, as well as social life, politics, and entertainment^5^.

Wearing of face masks has become an indispensable part of social daily life since the beginning of the COVID-19 pandemic^6,7^, yet mask-associated dry eye (MADE) becomes a new concern^8–10^. To date, however, there is little evidence on MADE after long-term wearing of face masks. Dry eyes may impact visual, physical, and psychological functioning; social activities, such as reading, watching, driving, and performing; and quality of life^14–19^. It is a progressive disorder characterized by disturbed homeostasis of the tear film^11–13^, even resulting in depression and increased economic burden^16,20^. However, little data is available regarding the prevalence and influencing factors of MADE in large-scale population studies. Therefore, we conducted a cross-sectional survey to investigate the association between wearing face masks and dry eye susceptibility in Chinese daily face mask wearers.

## Methods

### Study design and participants

This was an observational, cross-sectional, and descriptive study involving Chinese participants, conducted between January 29, 2021, and February 8, 2021, using an online survey. Ethical approval was granted by the Tianjin Eye Hospital Research Ethics Committee (Tianjin, China). All procedures were conducted according to the tenets of the Declaration of Helsinki^21^. None of the respondents were offered compensation or incentives to participate. Data analyses were designed and completed from February 14, 2021, to March 10, 2021. This study followed the Strengthening the Reporting of Observational Studies in Epidemiology (STROBE) reporting guideline for cross-sectional studies. The data were sourced from an online Internet platform (WeChat instant messaging app, Shenzhen, China) after distributing an anonymous nationwide online survey. And the informed consent was obtained from all subjects and/or their legal guardian(s).

### Questionnaire

A 19-item MADE questionnaire was drafted by a panel of experts from Tianjin Eye Hospital based on the survey of Boccardo and adjusted according to previous studies for assessing MADE particularly during the COVID-19 era^8,11^. This new questionnaire based on a national survey was named as the MADE-Q for the current study during the pandemic. MADE-Q contained questions on age, sex, education, mask wearing time, symptoms and frequency of dry eye (i.e., burning sensation, foreign body sensation, itching sensation, dryness, eye pain, grittiness, or irritation), history of dry eye medicines, daily reading time, outdoor time, and visual display terminals (VDTs) time. For symptom frequency, participants could select never (0 time per day), sometimes (0–4 times per day), or often (> 4 times per day). To facilitate a better response to the MADE-Q, the 19 questions were concisely constructed, and all symptoms were grouped into one single question. In adherence to the study design, the MADE-Q comprised 19 items representing the factors affecting MADE.

### Data management and statistical analyses

**Figure. 1:**
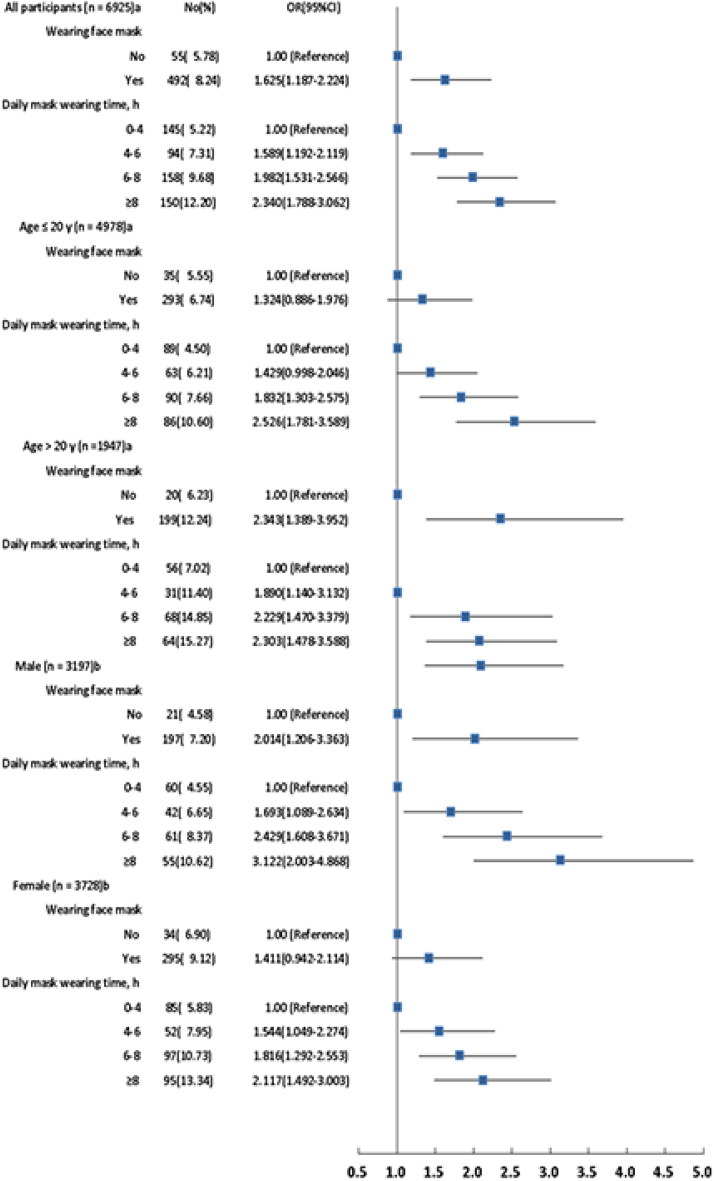
Forest plot of the association of wearing face mask and daily mask wearing time with MADE according to various populations and sex.

Data for sociodemographic characteristics and influencing factors were analyzed. The association of MADE with mask wearing was illustrated by a forest plot (Fig. 1). Odds ratio (OR) and 95% confidence intervals (CIs) were calculated with adjustment for different variables. We analyzed the data using SAS® software. All analyses were performed using SAS version 9.2 (SAS Institute, Inc., USA), Stata version 12.0 (Stata Corporation, USA), and Excel version Office 365 (Microsoft Corporation, USA).

### Meeting presentations

This study has not been presented at a meeting and is not under consideration for presentation at a meeting.

### Ethics approval

The Ethics Committee name: Tianjin Eye Hospital Ethic Committee. The study protocol number: 2021003.

## Results

### Questionnaire selection

**Figure 2:**
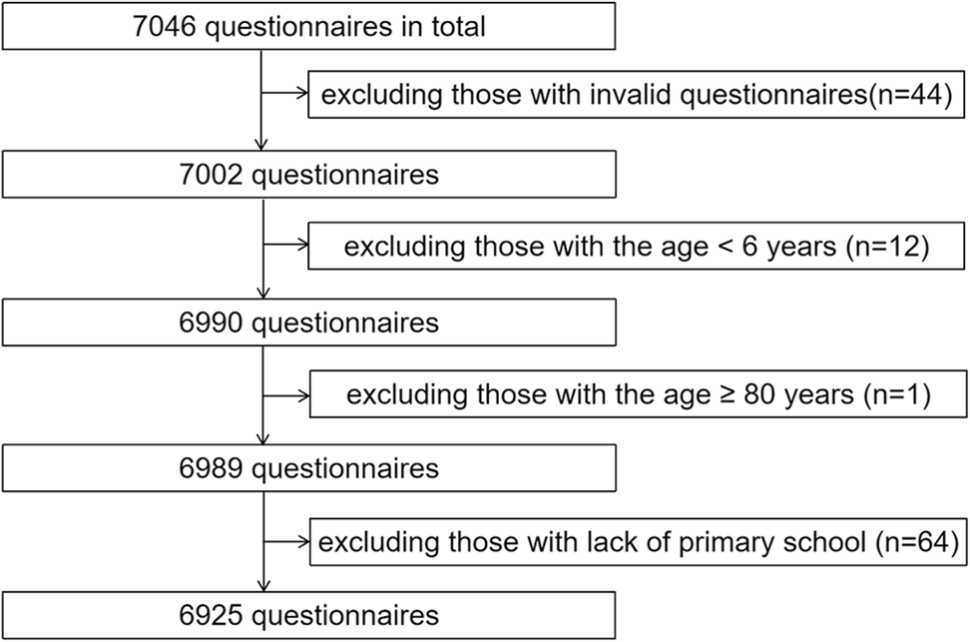
Flow chart of questionnaire selection.

The data collected from the online questionnaires showed the geographical location of the respondents. A total of 7046 anonymous online questionnaires were screened. Forty-four questionnaires were excluded due to incorrect data and 77 nonstandard questionnaires were excluded because they met the following criteria: uncorrected or unfinished answers for all the questions, age ≥ 80 years, age < 6 years, and lack of primary school completion. Finally, 6925 questionnaires were eligible for analysis (Fig. 2).

### Baseline characteristics of study population

The participants’ age ranged from 6 to 79 years (median: 14 years; interquartile range [IQR]: 12–26 years), and 6072 participants never experienced dry eye symptoms after wearing face masks; 419 participants sometimes and often experienced dry eye symptoms, and 128 participants experienced dry eye symptom aggravation. No change in dry eye symptoms was reported in 258 participants, while only 48 participants felt that their symptoms improved after wearing face masks. Regarding duration, 5973 participants wore face masks for ≥ 6 months. The overall rate of MADE incidence was 7.90%. Table 1 shows the demographic characteristics of the total study participants. Table 2 shows MADE information for the total study cohort.

**Table 1:** Baseline characteristics of the study participants.

Variables	Wearing of face mask				All	χ^2^	*P* value^a^
	No		Yes				
	N	%	N	%	N		
Age, years							
≤ 10	69	7.25	1177	19.71	1246	134.684	< .001
11–20	562	59.03	3170	53.07	3732		
21–30	48	5.04	403	6.75	451		
31–40	124	13.03	750	12.56	874		
≥ 41	149	15.65	473	7.92	622		
Sex							
Male	459	48.21	2738	45.84	3197	1.863	.172
Female	493	51.79	3235	54.16	3728		
Schooling							
Primary school	105	11.03	1784	29.87	1889	155.849	< .001
Middle school	344	36.13	1904	31.88	2248		
High school	187	19.64	799	13.38	986		
University or graduate	316	33.19	1486	24.88	1802		
Daily mask wearing time, h							
0–4	812	85.29	1964	32.88	2776	958.288	< .001
4–6	87	9.14	1199	20.07	1286		
6–8	40	4.20	1593	26.67	1633		
≥ 8	13	1.37	1217	20.38	1230

**Table 2:** The incidence of MADE in the study participants.

Variables	MADE				All	χ^2^	*P* value^a^
	No		Yes				
	N	%	N	%	N		
Age, years							
≤ 10	1159	93.02	87	6.98	1246	43.738	< .001
11–20	3491	93.54	241	6.46	3732		
21–30	394	87.36	57	12.64	451		
31–40	778	89.02	96	10.98	874		
≥ 41	556	89.39	66	10.61	622		
Sex							
Male	2979	93.18	218	6.82	3197	9.522	.002
Female	3399	91.17	329	8.83	3728		
Schooling							
Primary school	1758	93.07	131	6.93	1889	61.518	< .001
Middle school	2122	94.40	126	5.60	2248		
High school	913	92.60	73	7.40	986		
University or graduate	1585	87.96	217	12.04	1802		

### Data analysis

#### Mask wearing time and dry eye frequency

Regarding mask wearing time, 1964 participants wore masks for 0–4 h/d; 1199 participants, 4–6 h/d; 1593 participants, 6–8 h/d; 1217 participants, ≥ 8 h/d. There were significant differences in the incidence of MADE among participants with different mask wearing times (OR 1.306; 95% CI 1.221–1.397; *P* < 0.001). The incidence of MADE increased with longer mask wearing time.

Pre-existing dry eyes were present in 508 participants, while 853 participants had self-reported dry eyes after wearing face masks, resulting in an increase in MADE. Compared with those who did not have pre-existing dry eyes, those with pre-existing dry eyes were prone to have MADE, and there was a significant difference in the incidence between the two groups (OR 4.822; 95% CI 3.856–6.029; *P* < 0.001). The breakdown of the frequency of conscious dry eyes before wearing masks was as follows: 84.19% never experienced dry eye symptoms, 13.53% sometimes experienced symptoms (0–4 times per day), and 2.28% often experienced symptoms (> 4 times per day). There were significant differences among the three groups (OR 4.647; 95% CI 4.035–5.352; *P* < 0.001). Two-hundred -and -fourteen participants were treated with anti-dry eye drugs before wearing masks, while 6711 were not. There was a significant difference in the incidence of MADE between the two groups (OR 2.199; 95% CI 1.501–3.220; *P* < 0.001). Finally, 246 participants were treated with anti-dry eye drugs after wearing face masks, and 6679 were not. There was a significant difference in the incidence of MADE between these two groups (OR 5.405; 95% CI 4.044–7.223; *P* < 0.001).

#### Mask wearing pattern

In the total survey population, the pattern of mask wearing was divided into three categories: ill-fitting (score 1–5), less standard (score 6–9), and standard (score 10–11). In total, 450 (6.50%) respondents reported an ill-fitting pattern; 2394 (34.57%), less standard pattern; and 4081 (58.93%), standard pattern. There were significant differences in the incidence of MADE among the three wearing patterns (OR, 0.804; 95% CI, 0.702–0.920; *P* = 0.002). This study also combined ill-fitting wearing (score 1–5) with less standard wearing (score 6–9) as nonstandard wearing of masks (score 1–9) for analysis. There was a significant difference in the occurrence of MADE among those who wore masks in a nonstandard pattern and those who wore them in a standard pattern (OR, 1.429; 95% CI, 1.200–1.702; *P* < 0.001). A higher incidence of MADE was related with nonstandard wearing of masks.

#### Wear glasses or/and CLs

In the total study population, 2742 participants wore glasses, 132 participants wore CLs, 156 participants wore both glasses and CLs, and 3895 participants did not wear any eyesight correction. There was no significant difference in the incidence of MADE between the participants wearing CLs and those wearing glasses (OR 1.123; 95% CI 0.636–1.983; *P* = 0.689). The MADE incidence was higher in those wearing glasses and CLs than in those wearing CLs only (16.03% vs. 10.61%). It was also higher in those wearing only CLs than in those wearing only glasses (10.61% vs. 9.56%). The MADE incidence was higher in those wearing glasses than those with no eyesight correction (9.56% vs. 6.32%).

#### Dry and moist area

**Figure 3:**
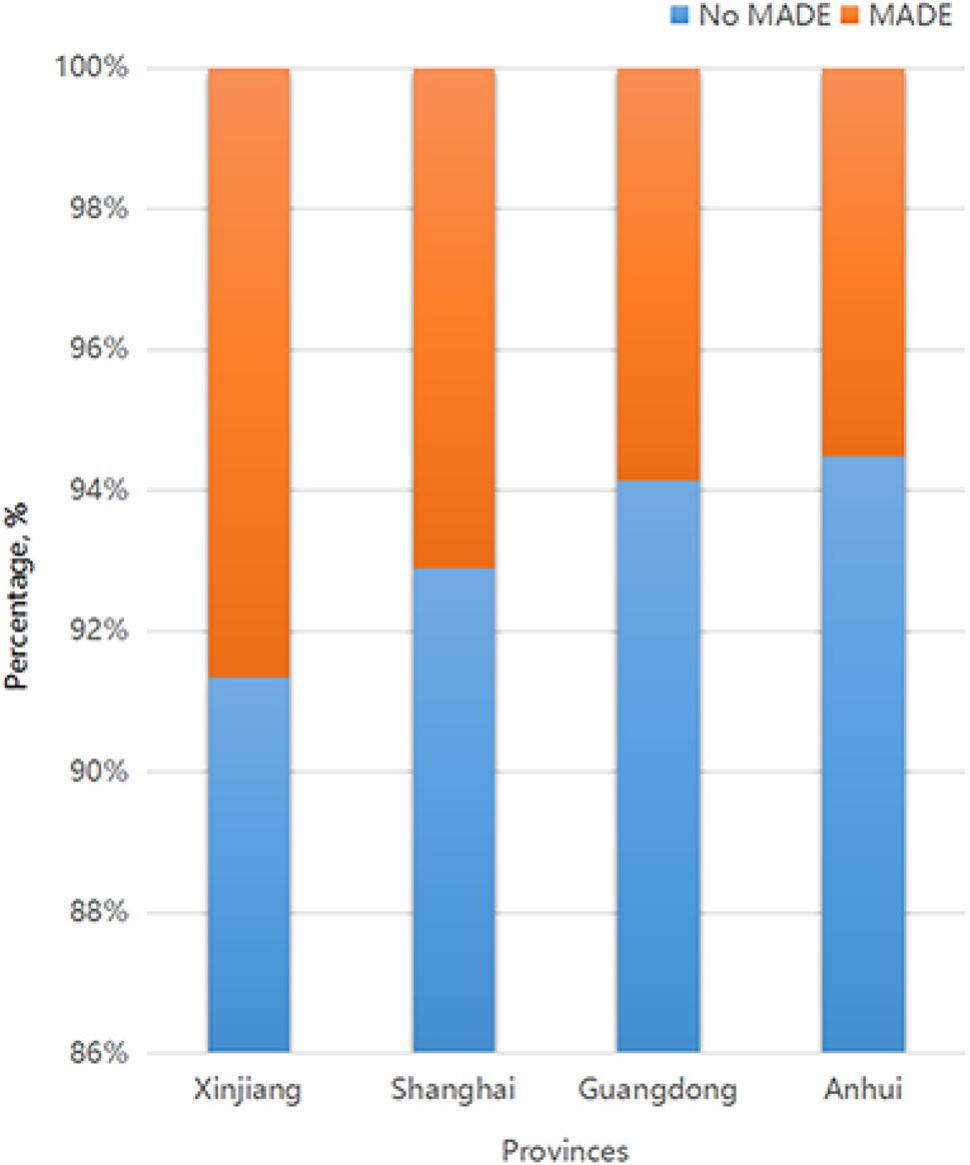
Analysis of MADE incidence at four different locations in China.

Four sites were selected for further analysis, representing dry and moist areas from the west to the east. The western site was Xinjiang, which represented the dry area, and the eastern sites included Shanghai, Guangdong, and Anhui, which represented moist areas. The site-specific distribution of MADE is shown in Fig. 3. Of those reporting MADE, 177 participants were from Xinjiang, 105 were from Shanghai, 20 were from Guangdong, and 79 were from Anhui. Significant differences were found in the incidence of MADE among these locations (χ^2^ = 13.354, *P* = 0.004). MADE incidence was significantly different across different locations and age subgroups (Table 3). MADE incidence was higher in the western area in the > 20-year-old population (OR 2.264; 95% CI 1.327–3.863; *P* < 0.001) than in the ≤ 20-year-old population (OR 3.612; 95% CI 2.100–6.212; *P* = 0.001).

**Table 3:** MADE incidence according to geographic location (West and East areas) and age.

Age, years	Place	MADE	χ^2^	*P* value	Model 1^a^ OR (95%CI)	Model 2^b^ OR (95%CI)	Model 3^c^ OR (95%CI)
		N (%)					
≤ 20	East	130 (5.13)	10.176	0.001	1.0 (reference)	1.0 (reference)	1.0 (reference)
	West	138 (7.47)			3.226 (1.957–5.315)	3.692 (2.226–6.123)	3.612 (2.100–6.212)
> 20	East	74 (10.35)	12.089	< 0.001	1.0 (ref)	1.0 (ref)	1.0 (ref)
	West	39 (19.50)			1.898 (1.208–2.982)	2.209 (1.383–3.526)	2.264 (1.327–3.863)

#### Age

Age was associated with the presence of symptoms in MADE (OR 1.199; 95% CI 1.119–1.284; *P* < 0.001) and with worsening symptoms (OR 1.019; 95% CI 1.013–1.026; *P* < 0.001). MADE incidence was higher in the > 20-year-old group than that in the ≤ 20-year-old group (Fig. 1). The associations between wearing of face masks, daily mask wearing time, and MADE incidence were investigated through age subgroups analysis (Fig. 1 and Table 3).

#### Sex

Overall, the study sample contained 3728 females and 3197 males. MADE was more common in females than males (Table 2). Female sex was a significant factor related with MADE (OR 1.323; 95% CI 1.107–1.580; *P* = 0.002) and with worse symptoms (OR 1.324; 95% CI 1.108–1.582; *P* = 0.002). The association between female sex and the wearing of face masks was significant (OR 1.411; 95% CI 0.942–2.114) (Fig. 1). MADE incidence was significantly increased with the prolonged daily mask wearing time in females.

#### Education

Regarding the education level, 1889 participants had completed primary school, 2248 had completed middle school, 986 had completed high school, and 1802 had completed university undergraduate or graduate education. There were significant differences in the incidence of MADE among people with different education levels (OR 1.277; 95% CI 1.183–1.378; *P* < 0.001). The educational level of university undergraduate or graduate was more associated with MADE than other levels.

#### Daily reading time

After wearing face masks, the daily reading time of 227 participants (3.28%) decreased by ≥ 2 h; 420 (6.06%), decreased by 1–2 h; 770 (11.12%), decreased by 0.5–1 h; 5180 (74.80%), remained unchanged; 117 (1.69%), increased by 0.5–1 h; 98 (1.42%), increased by 1–2 h; 113 (1.63%), increased by ≥ 2 h. There were statistically significant differences in the incidence of MADE among the groups (OR 0.822; 95% CI 0.749–0.902; *P* < 0.001). There was a significant difference in the incidence of MADE between those with decreased daily reading time and those with no change (OR 2.140; 95% CI 1.762–2.598; *P* < 0.001).

#### VDTs time

After wearing masks, the daily VDTs time (including online classes, watching TV, mobile phones, and playing games) was ≤ 1 h in 1975 (28.52%), 1–2 h in 2088 (30.15%), 2–4 h in 1561 (22.54%), 4–6 h in 738 (10.66%) and ≥ 6 h in 563 (8.13%). There was a significant difference in the incidence of MADE between different time periods of daily use of VDTs after wearing masks (OR 1.266; 95% CI 1.184–1.355; *P* < 0.001).

#### Outdoor time

After wearing masks, 749 participants had ≥ 2 h of reduced outdoor time; 974 participants had 1–2 h of reduced outdoor time; 1081 participants had 0.5–1 h of reduced outdoor time; 3833 participants had no change in outdoor time; 131 participants had 0.5–1 h of increased outdoor time; 72 participants had 1–2 h of increased outdoor time; and 85 participants had ≥ 2 h of increased outdoor time. There was a significant difference in the incidence of MADE between those with less outdoor time and those with no change (OR 2.027; 95% CI 1.692–2.427; *P* < 0.001), and between those with less outdoor time and those with more outdoor time (OR 1.747; 95% CI 1.081–2.824; *P* = 0.023).

## Discussion

This is the first study evaluating the relationship between face mask wearing and MADE prevalence in the general Chinese population. This cross-sectional survey was conducted in multiple provinces, with varying age and education level among the participants. The sample was representative of multi-ethnic rural and urban populations with different social-environmental statuses. The strengths of the study include a population-based design, a large sample size, and a standardized MADE questionnaire for assessing the related factors.

Many reports suggested that wearing face masks offered protection against COVID-19^7,22–24^. The World Health Organization (WHO) recommended the wearing of face mask by the general public during the COVID-19 pandemic, since its protective effect could have an important impact on transmission rates^23^. The ubiquitous use of face masks confers protective effects in many aspects of daily life, which even becomes a priority choice in some east Asian countries^24,25^. The types of face masks differ in different circumstances nationwide. Respirators are used to protect against COVID-19 in different settings, such as N95s, KN95s, and surgical masks, following the updated guidance of the National Centers for Disease Control and Prevention. The masks are made all by regular manufacturers with qualified standards and uniform specifications. The breather valve is not included in the face mask, and the masks are also required not to take off in most instances. However, few studies on the possible downsides of the widespread and long-term wearing of face masks have been reported. Hence, the exploration of the relationship between wearing face masks and dry eyes was inevitable and important for eye health and prevention, especially becuase it is likely that the worldwide population will continue to wear masks into the foreseeable future.

First, this survey identified significant relationships between face mask wearing and increased mask wearing time and MADE incidence (Fig. 1). The increased incidence of MADE due to the use of masks remained unaffected even after adjusting for confounders. This novel finding suggests that frequent wearing of face masks and longer daily mask wearing time are risk factors for MADE. Few studies have reported on MADE, which is a temporary condition induced by a sudden social and environmental change. Considering that MADE is induced by an etiology different than that of the dry eye disease, it is essential not to underestimate the disadvantages of mask usage, which could discourage the public from using them. Future investigations are needed to confirm and explain this finding further.

Moreover, different types of mask wearing patterns played a critical role in the MADE incidence rate. Nonstandard wearing of masks was significantly related with increased frequency of MADE. Another novel finding was that standard wearing of masks was a protective factor that prevented MADE. A recent study also suggested that correct use of face masks was important during the COVID-19^7^. It was essential to optimize face mask wearing, reduce the continuous mask wearing time, and ensure proper time intervals for mask removal.

We also found significant differences in MADE incidence among the four study sites. MADE incidence increased remarkably in the dry western area (Xinjiang) compared with the moist eastern areas (Shanghai, Guangdong, and Anhui) (Table 3). This finding suggests that dry environment is a risk factor for MADE occurrence and aggravation, which is consistent with the findings of a previous dry eye study^32^. According to Fagehi and colleagues^33^, dry eye cases were due to a dry environment which disturbed tear film stability. High prevalence of symptomatic dry eyes in Ghana was reportedly associated with windy conditions and less humid areas^34^. In fact, this novel finding of regional disparity in MADE incidence also revealed the association between MADE and exposure to a dry environment, indicating that environmental factors also played an important etiological role in MADE.

The incidence of MADE in the Chinese population was lower than that in Italians^8^. The difference might be attributable to age biases. The mean age in this study was lesser than that in the previous study^8^, and it was well known that dry eye occurrence was higher in the elderly than in the youth^12,13^. Our study was also consistent with the previous findings of a higher prevalence of dry eye disease in the elderly^17^. The initial TFOS DEWS epidemiology subcommittee report reviewed major international epidemiological studies and concluded that the prevalence of dry eye disease ranged from 5 to 30% in the ≥ 50 years age group^11,14^. In previous studies, the overall prevalence of dry eye disease based on the reported symptoms ranged between 14.4 and 24.4%^14–17^. In this study, relatively low representation from the older population was a concern. The reasons for this included less usage of mobile phones and a lower percentage and duration of face mask wearing by the elderly (especially retired people), which might have contributed to the low MADE incidence in this study (Table 2). There was indeed existing insufficient understanding in the collection of face masks related questions in 6–12-year-old children. The answers undertaken by the children were helped by their parents, who explained or admitted the question–answer process if necessary. Parents were asked to complete the questionnaire when children could not reply. The accuracy of answers to the questionnaire by the children might include deviation. Therefore, these questions were designed as simple, less quantity and easy to understand. They were firstly explained by their parents to the children and mostly or all answered by children themselves. This method was consist with other study^35^.

MADE incidence was higher in females than in males. This sex disparity was consistent with previous studies reporting a significantly higher prevalence of dry eye disease in females than in males^8,13,26,27^. Thus, females should be more cautious. Our study confirmed similar findings for both dry eye symptoms and MADE.

Approximately 5973 participants aged 6–79 years wore face mask for ≥ 6 months, including 4081 who wore masks in a best-fitting way. This differed slightly from a previous finding that 76.70% of the total sample population wore masks correctly^28^. Briefly, different mask wearing patterns were also related with MADE. This study emphasized on the importance of correct usage of face masks, as ill-fitting face masks might increase the risk of MADE; in fact, standardized wearing of masks could act as a protective factor.

Reduced daily reading time, less outdoor time, and shorter VDTs time were all significantly associated with MADE, which supported the new notion that these changes were due to wearing face masks in the COVID-19 era. This was contrary to previous findings that suggested that decreased outdoor time and increased screen time were more common during the COVID-19 pandemic^29–31^. However, we showed that reduced daily reading time, less outdoor time, and shorter VDTs time were associated with wearing of face masks, which was implemented in the general public during the pandemic. Educational level was also significantly associated with MADE. Higher education level was found to be a risk factor for MADE. Good compliance might be the reason for better educated participants accepting and supporting mask wearing. Additionally, wearing glasses and CLs was associated with MADE, and could worsen MADE outcomes. Wearing glasses and CLs might disrupt tear film stability, which was related with dry eyes. Future investigation is needed to identify the underlying mechanisms.

Overall, this survey investigated the prevalence of MADE among all age categories and found independent risk factors and protective factors across various etiological categories. It also underlined the importance of alerting the public about the risk of dry eyes induced by mask wearing. The online survey was a useful method for assessing self-reported symptoms, especially during this pandemic. In addition, the relationship between the optimum role of masks and MADE prevalence may need further assessment. Taken together, this study, based on more recent data, will reflect the potential trend of MADE incidence in the general public over time. We suggest that the increased risk of MADE should not be ignored in the current COVID-19 era.

### Limitations

Several study limitations should be noted. First, a caveat of the questionnaire was the lack of occupational categories and clinical confirmation of dry eyes. Second, it was an online survey which did not collect data on medical history, such as ophthalmic surgery history and systematic diseases history. Third, further investigation is needed to verify MADE incidence using a large sample size from multicenter studies. Fourth, a comparison of the occurrence of MADE in populations where mask use has identical use rates should continue to be presented in the future. Whether the MADE due to misuse or temporary use of the mask needs further study. Besides the type and wearing time of face masks as separated control groups, the climate, cultural background, cultural behavioral elements, race, occupation, and educational level should also be investigated in future study.

## Conclusions

In summary, this study provided novel data regarding MADE incidence as a consequence of wearing face masks in the COVID-19 era. Risk factors of MADE included longer mask wearing time, nonstandard wearing of face masks, dry environment, older age, female sex, higher education and less outdoor time. Pre-existing dry eyes and use of glasses and CLs could worsen dry eye symptoms. Standard wearing of face masks should not be ignored, as it is a protective factor against COVID-19. The optimum use of face masks in the health-care setting and daily life to mitigate their potential prejudicial effects are very important. Choosing the proper type of face mask, standardizing the fitting pattern of mask wearing, reducing continuous mask wearing time, increasing proper time intervals for mask removal, and lubricating the eyes with eye drops to help tear film recovery are recommended and may help in preventing MADE. People complaining about mask-induced eye discomfort should be made aware about MADE, and the clinical signs should be verified. Awareness about the possible risk of MADE should also be created.
